# The Casualty Surgery of Air Raids

**Published:** 1941

**Authors:** 


### THE CASUALTY SURGERY OF AIR RAIDS.

A MEETING of the Bristol Division of the British Medical Association was held in the Physics Lecture Theatre at the Royal Fort, University of Bristol, on Thursday, 10th July, 1941, with Dr. J. A. NIXON in the Chair.

A symposium was held on "The Casualty Surgery of Air Raids."

The CHAIRMAN : To-day we are having a similar meeting to that of last month in which there will be some talk on the casualty surgery of air raids, based upon actual experience. We have our local representatives who will speak from their own experience, with which some of us are familiar, and we are very fortunate in having two visitors who will speak from a wider field of experience : Mr. Rock Carling, who has seen casualty surgery in all parts of the kingdom, and Professor Ryle, who has had experience in some of the London raids.

### BLOOD TRANSFUSION.

Col. L. E. H. WHITBY : In opening this discussion, I should like to deal mainly with the subject of resuscitation, and to make two or three remarks about my own experience in Bristol in relation to equipment and amount, particularly of transfusion fluids which is required. I calculate 10 per cent. of all casualties require transfusion, whilst the average amount is from two to three pints, so that if one is receiving 100 casualties in a hospital one should lay down a stock of between 20 and 30 pints of transfusion fluid, and it is important that those stocks should be to hand ready for use by the resuscitation officer responsible for the work.

I should like to say first of all a word or two about shock in general—as to what is meant by the term "shock." It is a useful term which conjures up a picture of a person who is exhibiting lowered vitality as a result of injury, and the most striking manifestation is either circulatory embarrassment or collapse, which in clinical terms is represented most obviously by a reduction in the blood-pressure. There are a number of causes of that state, that is, it is not a definite clinical entity, it is only a train of symptoms, and it is essential for a reception officer to sort out the cases rather than merely to regard transfusion as efficacious for the treatment of every one exhibiting these symptoms. It has been found as a result of experience that the blood-pressure (particularly the serial blood-pressure readings) is the most reliable index of patients' condition, and the pulse, on the whole, is not nearly as reliable as was previously thought.

So far as the reception ward is concerned, it may be taken that an experienced reception officer at one glance can determine patients who should go to the resuscitation ward and those who can be sent to the general wards. That cuts down the amount of manipulation and examination of the patients which should always be minimal, because that kind of movement undoubtedly contributes greatly to their distress. So far as air raid casualties are concerned—and I say that with emphasis as distinct from battle casualties, and because our ideas on shock are largely influenced by the experience of the last war when we were dealing with a different type of material—they fall into two main classes: first, the neurogenic or psychogenic type, and secondly those who are suffering from a very definite reduction in blood-volume. There are other complicating factors such as fat embolism and so on, but the majority fall into those groups, and the treatment, of course, is different for each. The neurogenic and psychogenic, those who have circulatory embarrassment because of pain or because of fear, do not need transfusion, whereas those who have circulatory embarrassment because of blood-volume reduction necessarily do so, and the whole object of treatment in the second group is to restore by a quantitative replacement approximately the amount of fluid they have lost, and it is important that replacement should be made with a protein fluid.

In general principle, therefore, you have to sort out those who are likely to respond to conservative measures only, that is, rest, warmth, morphia, plenty of fluids, and most important, reassurance. The really frightened are greatly comforted by being told that they are not going to die, and this class have to be sorted out from those who definitely require transfusion, together with conservative measures.

We have learned several lessons about these cases in particular, and in relation to these two groups, the first being the great importance of the blood-pressure determination, and of the serial blood-pressure determination. If one is in doubt one should not be satisfied with a single reading, and after transfusion one is not satisfied unless the blood-pressure is good, so that there should be ample facilities for making serial blood-pressure observations on all patients. It is not necessary to have a battery of sphygmomanometers. All that is needed is a large supply of cuffs; the instruments can be plugged in to the points nearest the patient. The second lesson of great importance is that it is essential to know the nature of the injuries; superficially a person may appear to be uninjured, yet if he is fully examined it may be found that there is a small penetrating wound. That knowledge is essential in order that one does not fail to transfuse a person who should be transfused, and the best equipment is a large pair of carpetmaker's scissors. Nothing should prevent the slitting up of a person's clothes from head to foot if necessary in order to make a proper examination.

The third rule we have made is a corollary to the blood-pressure reading. There are people who can suffer the greatest reduction in blood-volume and not have an equivalent fall in blood-pressure. A

patient may have gross injuries and one would estimate from experience that his blood-pressure would be almost unrecordable : these are cases in which common sense dictates that he must have suffered a great loss of blood, and transfusion should not be withheld merely because the blood-pressure reading is not as low as might be expected. Anyone who is severely injured and who has obviously lost blood should have transfusion up to a pint, and this treatment should not be delayed merely because the blood-pressure has not fallen dangerously low. We have found oxygen of great value in any case which exhibits gross cyanosis, and there is much to be said for the only surgery that should be performed in a resuscitation ward, the treatment of any penetrating wound of the chest : it relieves the cyanosis and enables one to put the patient in a better position to withstand operation.

There is no doubt that once a person has been resuscitated adequately he should be operated on without delay, those whose blood-pressure is restored and general condition improved tend to fall away if they are not attended to surgically with fair speed. Resuscitation does not end in the resuscitation ward, we may restore a patient sufficiently for him to leave the ward and go to the theatre, but if he is severely injured he may require more transfusion in the theatre ; the movement from the trolley to the table and loss of blood at operation may undo some of the work previously done, so that in all such cases it is as well for the last act in the resuscitation ward to be the setting up of a bottle of fluid or plasma to go with the patient to the theatre. The rate can be speeded up without any delay supposing he suffers any deterioration whilst on the operating table.

I should like to show you two tables in order to illustrate some of the points I have been trying to make.

RESUSCITATION WARD RECORDS OF AIR RAID CASUALTIES

(Excluding further transfusions required in Operating Theatres.)

| | Case 1 | Case 2 | Case 3 |
|---|---|---|---|
| Calculated blood loss (c.c.) .. .. .. | 3,500 | 1,600 | 3,000 |
| Total fluid administered (c.c.) .. .. .. | 2,160 | 1,620 | 1,620 |
| Protein fraction (c.c.) .. .. .. .. | 1,540 | 1,155 | 1,155 |
| Per cent. of total lost fluid replaced .. .. | 62 | 100 | 54 |
| Blood-pressures :— | | | |
| Initial .. .. .. .. .. .. | un. | 50/35 | 35/5 |
| After first pint .. .. .. .. | 40/un. | 70/50 | 80/45 |
| ,, second pint .. .. .. .. | 60/40 | 85/55 | 95/60 |
| ,, third pint .. .. .. .. | 80/50 | 105/65 | 115/70 |
| ,, fourth pint .. .. .. .. | 105/55 | — | — |

The first table concerns three cases and enables me to make certain points. By means of laboratory examination it is possible to make a rough estimate of the amount of blood lost when a person is wounded. In the first case you will notice that the calculated amount of blood lost is 3½ litres, an almost incredible amount, one would hardly believe that a patient would survive, but it is one of the things we have learnt during this war that patients may lose even that large amount and still remain alive for a few hours. Anyone who lost that amount under battle conditions would probably never survive because of the lapse of time between receiving the wound and treatment. That is one of the essential differences between the casualties seen now and those of the last war. In that first case the blood-pressure was unrecordable ; it took four pints to get his blood-pressure to a reasonable level, which represented 62 per cent. of the fluid which he had lost as the result of hæmorrhage. It is necessary to replace something more than 50 per cent. of the volume lost by a protein fluid, and if this is done a reasonable result can be anticipated. The blood-pressure reading is the only reliable guide that the transfusion has been sufficient. One could make several points out of the table. One expects a rise in blood-pressure of from 10 to 20 mm. mercury for every pint of fluid transfused. There are reasons why the expected rise may not occur and it should put the transfuser on guard at once. If that kind of rise after each pint does not occur the commonest cause is continued bleeding from an internal source, one is merely pouring fluid into the vein and it is pouring out somewhat more slowly internally.

The rate at which the fluid is administered is of considerable importance. Most casualties are in reasonable health before they are wounded and one has not to be so particular about the slow rate as when transfusing an anæmic person in civil practice. No harm will be done to a patient whose blood-pressure is below 100 Hg. by the transfusion of one or two pints at a fairly fast rate. Rate sometimes causes rigors—not all rigors are due to incompatible transfusions—and if the rate is slowed down the rigor will disappear and everything will be satisfactory.

The fallacy of transfusiou with saline can be shewn experimentally*; an animal is bled until the blood-pressure falls from 160 mm. mercury to 20 mm., and this is followed by the transfusion of a number of fluids. Citrated blood raises the blood-pressure to a reasonable level and maintains it there ; plasma does the same, serum not quite as well; saline raises the blood-pressure to 80 or 100 mm. and then it falls off. A permanent effect is never obtained with saline, the reason being that it is excreted fairly rapidly. There are many who think that the provision of a blood transfusion service is unnecessary, that they achieved extremely good results with saline before, and I think that is true. Their observations are accurate, they did achieve good results, for the reason that if a battle casualty has survived his hæmorrhage his physiological equipment for the restoration of his blood-volume will have been brought into action, he will dehydrate himself and be in a poor way on that account only, and in such a case a pint or two of saline is a life-saving measure. That emphasizes again the essential difference between our casualties of to-day and the casualties with which people in my service may have to deal.

I have said a great deal about the importance of the blood-pressure and its value in assessing the condition of the patient and providing a guide for the sufficiency of transfusion. But the rule is not absolute,

* Buttle, G. A. H., Kekwick, A., and Schweitzer, A., 1940, *Lancet*, ii. 508.

the blood-pressure is not an infallible guide. A woman was admitted with gross injuries and her calculated blood-volume loss was somewhere in the region of 3 litres, one which should have given an unrecordable blood-pressure, but we found her blood-pressure was in the region of 90 mm. Hg. Common sense dictated that she should be transfused and it was done and it took four or five pints to restore her. We followed her through many weeks and when she was well again we found that she had a hypertension with a blood-pressure of 190 Hg. and had lost 100 mm. Hg., she was therefore in grave danger. A second case is that of the very young, people in the teens and twenties, whose first natural reaction to loss of blood is an intense vasoconstriction in an attempt to conserve such blood as is left. That vasoconstriction may be so great that it keeps the blood-pressure at a reasonable level despite the fact that they have lost much blood. Clinically they appear intensely pale because of the intense vasoconstriction, so that if in a young subject there are extensive injuries and a blood-pressure which is higher than one would expect and they are intensely pale the blood-pressure should be discounted to a certain extent because the blood-pressure will fall very rapidly. More lives will be lost from lack of transfusion than from the giving of it. If it is obvious from the injuries that the patient has suffered blood-volume reduction transfusion should not be delayed until the pressure has fallen to the danger level because if that is done many cases will be lost.

### ORGANIZATION.

Professor J. A. RYLE: I have been fortunate since the air raids started in gaining experience in several ways. First, by having the opportunity of living at a hospital where we have been fairly busy; secondly, with sitting with helpful colleagues on the Committee of the Medical Research Council concerned with the problem of shock and wounds, and, thirdly, by "picking the brains" of other people.

I propose to supplement Colonel Whitby's remarks by speaking of organization and duties of personnel. If these are not arranged properly the best individual work may break down. That seems to me to be a reasonable line to take because one does find places where there has been little or no air-raid experience, and they are prepared to work on preconceived notions which have had to be scrapped.

One has to talk on a certain basis on a certain sized hospital and I propose to talk on the conception of a city hospital capable of taking up to or over a hundred total casualties, and I want first of all to state what we have come to regard as a minimum personnel in such an institution. In smaller places and smaller hospitals and hospitals without students conditions vary and plans have to be modified accordingly, but I would say in a city hospital of considerable size and ready to take patients up to 100 or over, there should be on duty a medical officer in charge, or a medical superintendent, who has many functions, a surgical O.C., a physician, a receiving or admitting officer, an anæsthetist, residents, and a radiologist or radiographer, in addition to the nurses and sisters appropriate for the work. Clinical assistants, stretcher bearers, fire watchers and so forth are also needed. It is of some importance to know what proportion of the different types of cases there might be during a single night, and roughly the figures have worked out in most places as follows : 50 per cent. of the cases admitted will not have to go to the operating theatre, some will be fractured ribs, some just bruised, or scratched, some will be moribund, some will be excited, and 25 per cent. will probably have to go through the resuscitation ward.

The best way to deal with this consideration of the organization of this work is to start from the admission of the patient, and we will assume we are dealing with stretcher cases, although, of course, sitting cases come in as well. The receiving-room officer : during the last war it was generally conceded he had to be a man of considerable experience because he had to decide whether a case could be put on a train and sent to Boulogne or whether he had to be resuscitated, or whether he was ready for operation or would be in a short time and all that was done in a quick and hurried examination. Conditions are very different in city casualties ; we have not had convoys bringing large numbers at a time. One or two ambulances might arrive at a time, but there has not, as a rule, been the swamping of the reception-room and the decision has not to be made immediately and on the spot, and it is not essential that the receiving or admitting officer should be a man of special experience, but he should be sensible. A senior house man or a specialist surgeon might be willing to take it on. When an ambulance load is brought in all he has to decide is whether the patients shall go to the resuscitation ward or to the general ward, and of the walking cases he has to decide whether they are for operation or can be dressed and put in the rest room. He can deal with an ambulance load in from five to ten minutes, which includes giving the anti-tetanus injection and taking particulars. If the patient is bad he is sent to the resuscitation ward straight away and it is the duty of the almoner's clerk to obtain particulars later. The reception officer makes a superficial examination of the patient but does not turn him over or look for wounds, he more or less decides "resuscitation," or "general casualty" ward.

With regard to the resuscitation ward, it is an essential part of the organization and I only mention it because we have found hospitals who say that they are not going to have resuscitation wards but will distribute patients among the various wards. Most of us would not accept that as a reasonable plan. A resuscitation ward is essential where all the cases requiring this treatment can be gathered together, and it should be a ward in as safe and quiet a place as possible. The personnel of the ward should include as its chief a physician who is going to make that his main contribution to the air-raid problem and under him he will have at least one or possibly two residents, one of whom is expert at transfusion—in some hospitals there is a transfusion officer—a sister and an appropriate number of nurses.

It is usual to have the cradles all ready and at times when the raids occur frequently, the beds are kept warm all the time with the cradles in position. At Guy's we have always arranged that the senior surgeon does not operate unless the number of cases is small, we consider his job is the most responsible of all because he has to decide what is done to the patients and when; and when the resuscitation ward begins to fill and the patients are getting quiet and warm and the physician has had a look round then comes the surgical chief to begin to assess the degree of wounding of each patient. He dictates to his assistant and a board is put up at the end of the ward with the name of the surgeon who is to operate on each patient and when the operation is to be done. One can operate too soon or one can operate too late, and those decisions, as to the optimum time of operation, are best made between the physician who is there all the time and the surgeon who comes round and assesses the degree of recovery.

The surgeon-in-charge has the same function to perform in the general casualty ward, but naturally he attends to the resuscitation ward cases first of all. There are a lot of difficult problems one cannot go into here, such as, when should the patient be undressed, should they be left on the stretcher, and so on, but the only answer is that a decision must be made in each case. Sometimes they are so bad they must be left on the stretcher and lifted up on to the bed. An examination cannot be made without a large pair of scissors. The very able sister in our resuscitation ward reminded me of a "tip" when I told her I was coming here. "Tell them to have a large supply of triangular bandages." Often a little bandaging has to be done while waiting for a theatre and triangular bandages can be put on any part of the body very easily and they are easily removed and save manipulation.

Those are the chief duties of the surgeon-in-chief, he consults with the physician about chest, nerve, and head injuries, and between them they make the difficult decisions about the best time for operation.

All radiography should be done with a portable apparatus, it is much easier, and saves the patient additional moving and saves the stretcher bearers who are often having an active and difficult time.

With regard to the subsequent movements of the resuscitated patient, it is important to continue the observation and continue the treatment throughout and after the operation, and, working on that principle, our general plan is that all the bad cases are returned to the resuscitation ward from the theatre and not sent to the general ward. The patients who have come quickly out of their shocked condition are frequently sent back to the general casualty ward, but any case which is giving great anxiety is brought back to the resuscitation ward. Our ward contains twenty-six to thirty beds and we have not been over-pressed. We have taken as many as a hundred cases in night raids and seventy-five in day raids, but we have always managed to have sufficient movement to be able to keep our severe cases under the eyes of those who have seen them from the beginning. That continuity of observation is very important.

Just one more word on the question of continuity. It is only by close watching of the changes, almost from minute to minute, not from hour to hour, that decisions can be made. The operating surgeon cannot rush up to see his patients between operations, and therefore it is a division of labour between the surgeon-in-chief with the help of the physician and his transfusion officers. The operating surgeons may not see the patient until he comes on the table, and it is asking too much to demand that they should leave the theatre.

There is no need to separate the sexes in the resuscitation ward so that the staff is concentrated in one place. They learn to know each other, and the apparatus is all in one place. That is another answer to hospitals who say they do not need resuscitation wards. It is a vital necessity and there is no doubt that it saves lives and also contributes to knowledge. We have the good fortune, at Guy's, of having a whole-time research physician who takes one badly-shocked case and studies it in the most intimate physiological way, and he, like Colonel Whitby and others, is in the process of collecting valuable scientific observations which will advance our knowledge of what must be a complicated condition, more complicated now than in the case of military casualties because we have patients whose ages vary from a few months to ninety years of both sexes ; some are in uncertain health, they have been shaken or buried and are cold and their injuries are extremely varied. Sometimes there is no external injury at all, sometimes there is a penetrating wound. There is every mixture of pathology and that is why it cannot be a simple problem, yet I feel sure we shall crystallize information which will help us to say " we can transfuse " or " we can do the operation at such and such a time." In every case there is an optimum moment for operation, which can only be decided by close observation.

### RECEPTION OF PATIENTS.

Dr. P. Phillips : The reception of patients is a domestic matter for each hospital, depending upon its type of accommodation and layout. At Southmead Hospital we have a first-aid post, and to the question whether all casualties should go through this post I would say No. The serious casualties can be readily distinguished. If they were sent to the first-aid post, it would only congest its accommodation and delay the severely injured reaching immediate assistance.

The serious cases are, therefore, brought direct to a reception-room, which consists of a large ward—entrance at one end, exit at the other and two smaller side wards adjacent, with kitchen, store rooms, and stretcher-room. It is divided down the middle by a light screen, and about twenty beds provided on each side—one for men and the other for women and children. The beds are provided with radiant heat, cradles, blankets, hot-water bottles, etc., so that simple methods for resuscitation from primary shock can be started at once. Cases not requiring surgical operation can be moved on to the appropriate wards as soon as they are sufficiently recovered, thus liberating beds for new arrivals. The receiving ward has, therefore, served the function of resuscitation ward also, very serious cases being admitted to the two side rooms so that a minimum of handling and movement is necessary. The arrival of cases has been fairly quick after the incidents, starting usually with a small number and working up to a maximum after several hours. I should like to add a word about the good work of the ambulance services. Casualties have been brought in so quickly that the severely wounded are soon under treatment. Under battlefield conditions many of these would have died before reaching hospital or any effective treatment. Other points of importance are :—

1. System of recording admissions so that statistics are available at any moment for the Casualty Bureau. These are done in triplicate so that three copies are available immediately.

2. The belongings of patients have led to a considerable amount of trouble, and there have been cases of the looting of personal possessions already and for that reason we have evolved a system whereby a bag is attached to the bed for clothes and boots, and a small bag for jewellery, etc. It is important that personal belongings should be listed and a careful check made of them.

3. With regard to the casualties themselves we have a card system whereby different coloured cards are used with the general treatment advised, *e.g.*, immediate operation, resuscitation, and so on. These are tied to the patient and all details put upon them : the amount of morphia, the A.T.S. injection entered on the front by the receiving officer, and on the reverse side the sisters record the time these are given. These cards follow the patients whilst they are in hospital. We have one sister in charge of the anti-tetanic injections, and another giving morphia. They do nothing else, and in that way we have managed to make certain that no case needing it escapes an anti-tetanic injection.

The most amazing thing about patients on admission is their indescribably filthy condition, and any question of asepsis is impossible. An important point yet to be learnt is how to remove a patient's clothes quickly and painlessly. With the advent of coupons for clothing this is going to be a problem. Large and powerful scissors are certainly necessary ; we want tailor's shears to open up the seams without mutilating the garments.

The fortitude of patients is remarkable.

A woman was brought in with bad laceration of the face and forehead and the first thing she told me was that she had had a marvellous escape. During those few moments I noticed her arm was bruised and I asked how she got that and she said, "The first aid man did that, doctor. You see I was buried, he was walking about and I said, 'Excuse me, you are walking on my arm.'" That is an example of a Bristolian's fortitude.

With regard to personnel I feel that persons of some experience and judgment are required to pick out those needing operation, although in my experience rapid surgery has seldom been called for. Our practice has been not to give any one team too many cases to deal with, and for the surgeons to see the cases selected and later to have the final word about the order in which they are taken to the theatre.

A review of our actual casualties : with regard to mortality 5 cases were brought in dead, 27 died within twenty-four hours from severe and multiple injuries, and 25 died after a longer period. The mortality rate works out at 10 per cent. Sex of admissions : 61 per cent. men,

31 per cent. women, 8 per cent. children under 16 years of age. Types of cases worked out as follows :—

| | Per cent. | | Per cent. |
|---|---|---|---|
| Head injuries, involving damage to skull and brain | 18 | Facial injuries | 1 |
| Scalp and superficial head injuries | 12·5 | Jaw | 1 |
| Chest | 6·4 | Eye injuries | 2·6 |
| Fractured limbs | 17 | Shock (with no obvious injury) | 10·2 |
| Spinal injuries | 2·4 | Abrasions, contusions, lacerations and bruises | 21·5 |
| Burns | 1·2 | Cases associated with pregnancy | 0·5 |
| Abdominal injuries | 1·8 | Crush injuries | 3·9 |

With about three exceptions all the fractures were severe compound injuries.

One important point about special cases. A man was brought to the hospital with no injuries except to the cornea, and he had a prolapse on the iris on both sides. It seemed very important to get him to an eye specialist at once, and we sent him in the ambulance to the Eye Hospital, where within half-an-hour he was being operated on. He recovered his sight and has only a refraction error left which can be corrected.

Finally, in reviewing air-raid casualties as a whole, I should like to point out that our youngest patient was 1 day old, our oldest 98 years. In this total war we are not dealing with a restricted age group of fit young persons. The range of clinical material is much greater.

Our wounded include the very old and the very young, both sexes, those who were previously healthy or possibly previously diseased.

Hence, we must all remain students of these new experiences, ready at all times to modify our ideas and treatment as may seem best, in the interests and well-being of those unfortunate fellow citizens who have fallen victims to modern barbarity.

### OPERATIVE PROCEDURE.

Mr. G. M. FitzGibbon : I will not say anything very much about organization, but in addition to Professor Ryle's remarks I would like to say that seeing the cases before operation has points and I am sure that one has learnt that there is not, in the majority of cases, a desperate urgency to deal with them. They have been benefited by fluid, rest, warmth, and during a good many of the raids when Professor Short and myself have been at the Infirmary we have managed to get round to see the majority of cases before we start operating.

With regard to the order in which they come to the theatre we have left that to the senior resident officer, Miss Fox, who has proved very able indeed in selecting cases and sending them down. She patrols the wards regularly with a complete list of cases, and from time to time she visits the doubtful cases to see whether they are deteriorating or improving, and at intervals she comes down to report that such a case is deteriorating or improving, the result being that we have seen the bulk of the cases before they come to the theatre.

We do not necessarily earmark cases for ourselves, whoever happens to have finished takes the case which is waiting, and if we have seen them previously we know sufficient about them to get on with it.

Dr. Phillips has referred to the mentality of patients ; they really are extraordinary, the unquestioning confidence which they have in the hospital and its treatment is amazing and their patience and fortitude is astonishing. On the whole I find they make most excellent subjects for operation, never questioning anything and accepting anything that comes to them.

We have had an enormous amount of very valuable assistance from Colonel Whitby and his assistants, and we have been extremely thankful for their work, they have undoubtedly saved the lives of many of the casualties.

It is extraordinary what can be done. I had a man injured in the daylight raid, a man of 60, with a leg shattered and an injury to his arm. He had an unrecordable blood-pressure when he came in—it was said to be about 20—and clearly he was going to die fairly rapidly. He had four pints of plasma and one of blood, and his pressure rose to 100. I did an amputation above the knee and dealt with the wound in his arm, and in two days he was sitting up reading the paper and made an excellent recovery. He would have perished within an hour or two if he had not had a transfusion.

I should like to emphasize what Colonel Whitby said about the danger of young adults who are able to compensate by vasoconstriction and give an entirely false clinical picture. They have a good pulse and their physical condition is not too bad. One may be tempted to start operating and the condition rapidly deteriorates and will cause anxiety in a very short time.

As regards actual operative procedure it is exceedingly difficult to give you any extensive picture of the surgery of air raids, but I should have said that a high proportion differ in no way from ordinary civil traumatic surgery, running down cases and industrial accidents, and present the same clinical picture. There is no special magic about it. The difference in some cases is where there are penetrating wounds with flying bits of bomb splinters and secondary missiles—I removed a piece of motor-bicycle from a man one night. It is difficult to give a wide picture of the different operative procedures. One of the most useful things is to talk about the cases one finds most difficult to cope with to see if anyone has suggestions or comments to make as to how they should be dealt with.

The type of case I have found difficult is the multiple pepper wound. Patients come in literally smothered with dozens of small puncture wounds and, as I think one of the speakers has pointed out, some of them may be of the greatest importance. It is astonishing what may lie behind a small penetrating wound of the abdominal wall and one wastes a lot of time exploring little abdominal wounds, but occasionally one finds something that makes it worth while.

So many of the patients do not complain. The most astoundingly severe wounds may be present without any complaints of pain, or a frank denial that they have any other injury so that it is worth exploring a wound as one gets surprises now and again.

Another case is the abrasion. The incredible filth of some of these is very striking and there are large superficial areas with dirt ground into them. If they are left to heal they leave scars afterwards, they are second degree injuries of the skin, but they present a difficulty as to the best means of cleaning them. I was taught to use a nail brush on them under a general anæsthetic with a piece of soap, and that is the only satisfactory method I have found in that type of injury.

Another type of injury that I find difficult is a penetrating wound which does not go through a limb. It may go three-fourths of the way and clearly if a *débridement* is to be done that is worth while it does involve considerable risk to nerves, arteries and vital structures, and I have resorted to making a counter incision on the other side and trying to make a conical wound, the two apices meeting at the centre point, and in that way one gets a track which is relatively clean. This is open to the objection that it opens up tissues that are not injured, but one has a fairly clean track. *Débridement* should be done with scissors. I have been most impressed with the effects of spraying or covering the raw surface of a wound with sulphanilamide powder, and there is considerable evidence to show that both experimentally and clinically the administration of the drug in this way is highly beneficial and has contributed greatly to a reduction in sepsis. Sulphanilamide powder is the best to use because of its high degree of solubility, and one can be satisfied that it has got into all the corners and crevices of an uneven wound and is better than the other drugs in this respect. There is plenty of evidence that it does not interfere with granulation or repair and healing goes on normally.

The powder is difficult to spray because it tends to coagulate into lumps and clots, it tends to pick up moisture. I saw a spray a few days ago in Sir Harold Gillies' clinic in use. [Here Mr. FitzGibbon showed the spray and demonstrated its use.] It consists of a piece of copper piping soldered across the lid of a glass box, with a piece of piping down to the bottom of the bottle and a hole into the inlet pipe. The bottle stands in the theatre until it is empty and at each operation the sister has a length of sterile rubber tubing and if sulphanilamide is needed the surgeon sprays it into the wound. Rubber dust caps are fitted at the end of the tube when it is not in use. Sir Harold Gillies uses sulphanilamide in considerable quantities, and it does not interfere with the healing of wounds in any way.

I have only seen two cases of gas gangrene, neither of them fatal, neither had had sulphanilamide or sulphapyridine in their wounds. The first was a punctured wound of the calf and I saw him three or four days after operation. His general condition was poor and I did not think he would stand an amputation, and I also did not think he would be clear if one did amputate. Several incisions were therefore made and obviously affected parts cleaned up, the wounds were packed with sulphapyridine and he was given serum. The next day he was about the same, on the second and third days further incisions had to be made and the condition flared up, but he got on top of it and improved. On the ninth day he had a venous hæmorrhage and had to lose his leg, but he was in a much better condition and in spite of an obviously gross and grim infection he did not die.

The second case was one that I did not do personally, it was a minor injury of the tibial region in a woman and next day there was intensive bronzing of the skin round. In view of my previous experience I simply carried out an extensive excision of the damaged skin and muscle, packed it thoroughly with sulphapyridine and gave her serum. She made a perfect recovery and I was able to skin-graft the area. She did not lose her leg.

I have only operated on two chest wounds, one because it was a progressive hæmothorax, a wound over the lower lung. I opened the chest, found a lacerated wound, sutured it, emptied out the clot, put in a pipette tube ; it had suction drainage and made a straightforward recovery. The other was a penetrating wound ; it missed the liver and went through the diaphragm and into the base of the right lung. I started at the wrong end, of course, not realizing that the chest was damaged. I followed the track which went through the diaphragm, I opened the chest, closed the wounds, put in a pipette tube and he made a complete recovery. I wonder whether we ought not to empty cases of hæmothorax, there is more danger of infection than one realizes, and I have a strong feeling that it is the right thing to do and up to now I have done it by putting in a tube and keeping them empty by suction.

On the subject of burns I feel quite sure that we have tanned too many burns. It is exceedingly difficult to realize the extent to which necrosis will result. The burn may look like a second degree and suitable for tanning, but a number of cases continue the necrosis underneath the tan and it is found subsequently that the tan is resting on granulation tissues and not upon healing epithelium and one is confronted with two of three awkward weeks getting tan off and then a skin graft. Only those causes in which we are absolutely satisfied that the burn is only skin deep should be sprayed. Necrosis and damage is increased by tanning an area in which the burn has gone deeper than the full thickness of the skin.

On the question whether we can improve the surgical facilities, I feel life might be a little simpler for the surgeon if he did not have to work in so many places, one does one case here and another somewhere else, and the amount of time spent in getting round to one or two cases is amazing. I should like to evolve some system whereby we could concentrate our work more. Then with regard to continuity of treatment : we have suffered rather badly from evacuation of casualties when they have been operated upon, and we have not beds within a short distance of the city where one can continue treatment of these cases. I think this is a criticism which is recognized and some steps are being taken to remedy it.

MR. E. ROCK CARLING : I think I will make a few comments on some of the things which have already been said and will take up Mr. FitzGibbon's last point about the organization of our work in conditions of heavy raiding. The fundamental principles of surgery are quite unaffected by externals like war, but the organization of our work in hospitals must be very different. I take it that a busy hospital in the ordinary event takes in in 24 hours perhaps 5 or 10

or even 20 cases and of those a comparatively small proportion need immediate serious treatment, but when you take in 50 or 60 or 100 within the space of two hours it is perfectly obvious that the organization which is satisfactory for the one type of admission is not satisfactory for the other.

When Colonel Whitby was speaking he mentioned the necessity of operating when the patient was ready. It not infrequently happens that a number of patients reach the optimum moment together, and it is very important that there should be available during the course of heavy raiding enough surgeons to supply all the available tables and to keep those tables supplied, sometimes over a very considerable period. It is comparatively easy when you have a raid one night and work during the following twelve or twenty-four hours, but it is by no means so easy when you have had continous raiding and have to work for many nights in succession. This means that we surgeons must give up any idea during conditions of raiding that we have any beds of our own or that we can select cases for our wards. All the beds of every hospital are the property of the public who require them, not of the surgeon or administration. It is of the utmost importance that during a raid there should be a surgeon or physician or both in supreme control.

Colonel Whitby's remark that the reception officer could see at a glance which patient must go to the resuscitation ward must be true, because throughout the country the untrained or partially untrained stretcher party leader has in fact succeeded in sending the serious cases to hospital and the minor cases to first aid posts. It puzzled me for a long time to know how this was done, but it seemed to be that every case which can help itself goes to a post and the helpless cases to hospital. About the patients who appear to be extremely well and who nevertheless are in danger because the initial high blood-pressure is going to fall away in due course, I think I am interpreting the opinion of the majority of surgeons when I suggest that these patients should be operated on before the moment arrives at which their blood-pressure will fall, but that transfusion must take place in the theatre and continue afterwards. These cases are ready for operation soon after they are admitted.

At one hospital the opinion was expressed that the raids were the affair of the house staff and not of the senior staff. I entirely disagree with that, certainly the most experienced members of the staff should be present during a raid. Every possible preparation should be made in the theatre for resuscitation and transfusion in the theatre for severely injured cases with comparatively little shock. Professor Ryle is in the happy position of having enough beds to enable his serious cases to be returned to the resuscitation team, but in the vast majority of hospitals only ten or twelve beds can be afforded for resuscitation purposes and in that event it is impossible to have the serious cases returned to the resuscitation room. Transfusion and the other methods must be continued in the general ward.

I agree with Dr. Phillips that the organization must be suitable for the particular conditions of every hospital. There are some where it is impossible to find a satisfactory resuscitation room without sacrificing a large number of beds, but 100 beds perfect for their purpose are better in conditions of heavy raiding than 200 so crowded that raid conditions lead to muddle. It is the universal experience that we must not rush and to make haste slowly is without question the right procedure.

I also agree with practically every word Mr. FitzGibbon said. It is very important that a surgeon should consult with the physician in charge of the resuscitation ward as to the most appropriate moment for operation and the order in which the cases should be sent to the theatre. There is this corollary, that if the surgeons do not see their cases very careful records must go to the theatre with the patient. It is extremely humiliating when an abdominal operation has been performed and one or two wounds on the limbs attended to, to discover that there is a penetrating wound which is traversing the edge of the spinal column and damaged a couple of nerves *en route*.

I must endorse what has been said about the importance at the appropriate moment for complete undressing and very thorough examination of the patient. That must often be postponed until resuscitation has proceeded a long way, but it must be done before the patient goes to the theatre.

It is true that a great many of these injuries are like those seen in civil life. My experience with regard to pepper wounds is that they take care of themselves and it is not necessary to spend a great deal of time in going over thirty or forty tiny wounds, but there is nothing more dangerous than an innocent-looking penetrating wound. A first aid doctor said that he had on a certain night done up many small insignificant wounds of the scalp. "Yes," said the surgeon who had received these cases, "you sent 11 in one night, 4 had foreign bodies in the brain and the other 7 had depressed fractures." Penetrating wounds are terrible pitfalls, very small fragments may do enormous destruction.

I hate the word "*débridement*." What we should have said long ago was that a wound should be laid widely open, because nobody knows how far the cut ends of severed muscles may retract. I have looked on at operations and longed to say, "For goodness sake, lay those two wounds into one and clear up that dead muscle or your patient will die of gas gangrene," and in at least one case I had almost to say it to a colleague of my own standing. Lay your wounds widely open until you can get to every possible recess.

With regard to abrasions a number of surgeons have resorted to scrubbing, but it was sometimes accompanied by very severe shock. Some people have used liquid paraffin as a cleaning agent and it is worth trying.

No surgeon would condemn Mr. FitzGibbon if he made a further incision in a penetrating but not traversing wound in order to ensure a clean wound. It is the only way to do so.

He said that sulphanilamide was of great value and that there was ample evidence of that. I am not quite sure that there is scientific evidence of it, but I will go this far and say I shall be extremely sorry if I have a large wound on my soft parts and the surgeon operating on me does not use it. Those soldiers who were evacuated from Crete without any treatment at all except some sulphanilamide put upon the surface of their wounds arrived in Egypt in an extremely satisfactory condition. Not more than 15 grammes should be put into wounds, some people think 5 is sufficient, but I think something like 10 grammes and possibly more is needed in big wounds. Some surgeons have begun to use an emulsion of sulphanilamide in order to make sure of it getting into the wounds and others have employed it in a vaseline base, but the proper base has not been discovered.

With regard to the cases of gas gangrene which recovered where amputation was not advisable at the time, I wonder if Mr. FitzGibbon has had any experience of zinc peroxide. Mr. Tudor Edwards is the great exponent of the use of zinc peroxide for gas gangrene in this country.

A hæmothorax should certainly be kept empty.

I cannot go into the whole story of coagulation of burns, but I think the vast majority of surgeons would agree that we have been trying to teach the medical public that coagulation is the life-saving method of treating burns and now we say, " You must not use coagulation." It has been put forward by the plastic surgeons that in third-degree burns where the skin is destroyed in toto, they should not be coagulated. They do sometimes heal perfectly under coagulation but they heal with the loss of the amount of skin that has been destroyed, the healing takes place by the drawing of the skin together to fill the part that has been lost. It does not epithelize over the whole of the lost surface and for that reason and because tanning is a bad preparation for future grafting the plastic surgeon would like every third-degree burn treated by any means other than coagulation. If one could be certain that a burn was third degree the answer would be simple, but every burn which is third degree is also first and second. If there is some part of a burn which is third degree the use of coagulants is precluded.

I must go back to the question raised by Mr. FitzGibbon on the question of operating at many places and the continuity of treatment. It must be extremely difficult to arrange this because we are not doing casualty work all the time, we are continuing our peace-time work at the same time. It does seem preposterous that we should spend a large amount of time doing work in different places instead of concentrating at the hospital where we do our casualty work, and the hospital to which those cases are evacuated. I do not see how any body—the E.M.S. or the Ministry of Health—can make such changes, it can only be arranged locally. It is not beyond the wit of man to do so and it would work to the advantage of the public. Odd little things bring about great changes and I suppose it is quite possible that a shortage of petrol may effect the change which we are unwilling to arrange for ourselves. If so, it will be the only good thing one knows about petrol !

Mr. K. H. PRIDIE : The cases come so very quickly from the scene of the incident that it seems unnecessary to put forward any complicated plan for a 10 minutes' ride. The casualty has to be taken off the ambulance for examination, taken to the resuscitation ward, examined again, taken to the theatre and unbandaged again. We do not see the Thomas splint used for first aid work because it is impracticable, but I should like to make some criticisms on the first aid work. Use is not made sufficiently of a firm bandage over wool.

I have only once seen a case where a tourniquet definitely saved a life, I have many times seen a tourniquet causing unsafe venous conditions. The dressings issued to the wardens are too small for the exceptionally large wounds we see in air-raid work. The Army authorities have a large, firm bandage which is much better. More use should be made of Liston's long splint, it is most useful for anything in the lower limb. The people at the point of the incident should straighten the limbs. I saw a case the other day of a femur sticking out and the man sitting on his own leg, it was completely crushed. Longitudinal traction should be put on the limb and a firm bandage over wool will stop any hæmorrhage and the doctors should go to the scene of the incident and supervise this work.

The doctor at the incident should put a label on the patient saying whether it is a compound fracture, so that on reception there is no need to take off the firm bandage which lets the life blood flow away. Much hæmorrhage is caused which should never have happened by the removal of a firm bandage. The bandage should not be disturbed until the patient is in the theatre. Patients should be treated by a doctor at the incident, we should get better first aid if that was done and I hope an experiment will be tried in this respect. It is not so dangerous as it sounds.

The CHAIRMAN said that Mr. Gordon Paul and Mr. Berry Haycraft had not been able to attend the meeting. He proposed a very hearty vote of thanks to the contributors to the discussion and particularly to Professor Ryle and Mr. Rock Carling who had come great distances to take part in the symposium.

The vote of thanks was accorded by applause and the meeting terminated.

